# Bee venom promotes exosome secretion and alters miRNA cargo in T cells

**DOI:** 10.1515/biol-2025-1180

**Published:** 2025-10-03

**Authors:** Ziyan Cui, Zegao Zhou, Ziyan Sun, Jiayue Duan, Runtian Liu, Cheng Qi, Changqing Yan

**Affiliations:** Department of General Surgery, The Second Hospital of Hebei Medical University, Shijiazhuang, China; Clinical College of Hebei Medical University, Shijiazhuang, China

**Keywords:** bee venom, T cells, exosomes, miRNA, high-throughput sequencing, cancer

## Abstract

Exosomes are important mediators of intercellular communication, primarily through the transfer of miRNAs. However, the mechanisms regulating exosome secretion and miRNA cargo loading in T cells remain incompletely understood. In this study, we investigated the effects of bee venom (BV), a natural compound with known immunomodulatory activity, on the exosomal miRNA profile in human Jurkat T cells. The non-cytotoxic concentration of BV (2 μg/mL) was identified using the CCK-8 assay. Exosomes were isolated from BV-treated and control cells and characterized by transmission electron microscopy, Western blotting, and nanoparticle tracking analysis (NTA), with NTA also used to quantify particle concentration for assessing changes in secretion levels. High-throughput sequencing identified 74 differentially expressed miRNAs (44 upregulated, 30 downregulated), which were validated by quantitative real-time PCR. Functional enrichment analyses (gene ontology, kyoto encyclopedia of genes and genomes, disease ontology, Reactome) revealed associations with pathways related to neural development, cell cycle regulation, and tumorigenesis. BV treatment significantly promoted exosome release and selectively altered miRNA cargo. These findings suggest that BV modulates T cell–derived exosome output and composition, providing mechanistic insights into how it may influence immune-related signaling pathways.

## 1 Introduction

Exosomes are small, lipid bilayer-enclosed extracellular vesicles (30–150 nm in diameter) that are secreted by nearly all cell types and present in various bodily fluids [1]. These vesicles serve as crucial mediators of intercellular communication through the transfer of biologically active molecules – including proteins, lipids, mRNAs, and microRNAs (miRNAs) – from donor to recipient cells [2]. Among these cargo molecules, miRNAs are of particular interest due to their critical function in regulating gene expression post-transcriptionally [3]. Exosomal miRNAs have been implicated in numerous physiological and pathological processes, such as cell proliferation, differentiation, immune modulation, neurogenesis, and cancer development [4,5,6]. Due to their stability and specificity, exosomal miRNAs have emerged as potential biomarkers [7].

T lymphocytes, especially Jurkat T cells used as a model for human T cell leukemia, are central players in adaptive immunity [8]. These cells not only exert immune responses through receptor-mediated signaling but also secrete exosomes that participate in shaping the immune microenvironment [9]. T cell-derived exosomes can influence dendritic cells, macrophages, B cells, and even tumor cells through the delivery of regulatory miRNAs [10,11]. The composition and function of these exosomes are dynamically influenced by environmental cues and pharmacological agents [12]. However, the precise molecular factors that enhance exosome secretion and miRNA cargo packaging in T cells are not fully understood.

Bee venom (BV), a complex natural secretion from the venom glands of Apis mellifera, contains a variety of biologically active peptides and enzymes, including melittin, apamin, and phospholipase A2 [13]. It has long been used in traditional medicine and has more recently been investigated for its therapeutic potential in cancer, arthritis, and neurodegenerative diseases [14–16]. BV exhibits diverse biological effects, such as anti-inflammatory, cytotoxic, and immunomodulatory activities. Previous studies have shown that BV can influence immune cell activation, apoptosis, and cytokine release [17,18,19]. However, whether BV can regulate the biogenesis and molecular content of exosomes, particularly in T cells, remains largely unexplored.

In this study, we hypothesized that BV, a natural compound with known immunomodulatory effects, modulates exosome secretion and miRNA cargo composition in T cells. To test this, we used human Jurkat T cells to assess the impact of BV treatment on exosomal output and miRNA profiles. A non-cytotoxic dose was selected, and exosomes were isolated and characterized using established techniques. High-throughput sequencing and bioinformatic analysis were then performed to explore BV-induced changes in exosomal miRNAs. The aim of this study is to provide new insights into how BV may influence immune communication via extracellular vesicle regulation.

## 2 Materials and methods

### 2.1 Cell culture

T cells (JurKat, Pricella, CL-0129) were cultured in RPMI-1640 medium (Hyclone, SH30809, USA) supplemented with 10% exosome-depleted fetal bovine serum (ViVaCell, C3801-0100) and 1% penicillin-streptomycin (Gibco, 15140112, USA) in a humidified incubator at 37°C with 5% CO_2_. Upon reaching the logarithmic growth phase, cells were divided into two groups: the BV-treated group (BVT), treated with BV (North China Pharmaceutical Co., Ltd, China) at concentrations of 2, 4, 6, 8, and 10 μg/mL; and the control group (CT), treated with an equivalent volume of phosphate-buffered saline (PBS, Gibco, 10010023). Commercial lyophilized BV was reconstituted in sterile PBS, filtered through a 0.22 μm membrane, and either used immediately or stored in aliquots at –20°C, protected from light. All experiments were performed using a single batch of BV (Batch No.: 1EFNE30201) to ensure consistency. In this study, all experimental conditions were tested in triplicate using independent biological replicates.

### 2.2 CCK-8 assay to determine non-cytotoxic BV concentration

Cell viability was assessed using a CCK-8 assay (Solarbio, 40203ES, China) at 0, 24, 48, and 72 h. Treated cells were seeded in 96-well plates at a density of 1,000 cells per well in 100 μL of medium. At each time point, 10 μL of CCK-8 reagent was added per well. Control wells received medium with CCK-8 only. After a 1 h incubation at 37°C, absorbance was measured at 450 nm using a microplate reader (Hui Song, MB 530, China). Cell proliferation rates were calculated from optical density values to generate growth curves. The BV concentration that did not significantly inhibit T cell proliferation was selected for subsequent experiments.

### 2.3 Exosome extraction and purification

Following identification of the non-cytotoxic BV concentration, both T cell groups were cultured further, and supernatants were collected 48 h post-treatment [20]. Exosome isolation was performed based on a modified Geboloğlu protocol [21]. Briefly, supernatants were thawed at 37°C and sequentially centrifuged at 2,000 × *g* for 30 min and 10,000 × *g* for 45 min at 4°C to remove cells and debris. The supernatant was then filtered through a 0.45 μm membrane and ultracentrifuged at 100,000 × *g* for 70 min at 4°C (Hitachi, CP100MX, Japan). The pellet was washed with PBS and subjected to a second ultracentrifugation under the same conditions. Exosome pellets were resuspended in 300 μL of PBS for further analysis and stored at –80°C. Exosomes derived from BVT and CT cells were denoted as BVT-exo and CT-exo, respectively.

### 2.4 Exosome characterization

A drop of exosome suspension was applied to a copper grid, negatively stained, and imaged via transmission electron microscopy (TEM, Hitachi, HT-7700) to assess morphology and size. Nanoparticle tracking analysis (NTA; ZetaVIEW, Particle Metrix, Germany) was used to measure particle size and concentration. The Stokes–Einstein equation was used to estimate the diffusion coefficient and hydrodynamic radius, and concentrations were adjusted for dilution. Exosomal proteins were quantified using a BCA assay kit (Thermo Fisher Scientific, 23227, USA), and Western blotting was performed following SDS-PAGE. Proteins were transferred to PVDF membranes, blocked for 1 h, and incubated overnight at 4°C with primary antibodies against CD9 (Boster, BM4212), CD81 (SAB, 41779), CD63 (Abclonal, A19023), and TSG101 (Abcam, ab125011). Secondary antibodies (Invitrogen, 31460) were applied for 1 h at room temperature. Signals were visualized using a chemiluminescent imaging system (CLINX, Chemiscope 3000mini).

### 2.5 miRNA sequencing

Total RNA was extracted from T cell-derived exosomes using the miRNeasy Mini Kit (Qiagen, 217004, Germany). RNA concentration was measured using a Quantus Fluorometer (Promega, USA), and the integrity of small RNAs was assessed using the Qsep100 automated capillary electrophoresis system (BiOptic, China) in combination with the NR1 High Sensitivity RNA kit (BiOptic, C105211). Small RNA libraries were prepared using the QIAseq^®^ miRNA Library Kit (Qiagen, 331505) and sequenced on the Illumina NovaSeq 6000 platform using a paired-end 150 bp strategy. Each sample yielded approximately 12–22 million raw reads on average. FastQC (v0.12.1) was used to evaluate the quality of the raw reads, and fastp (v0.23.2) was employed to trim adapters, remove low-quality reads (Phred score <20), and filter out poly-N sequences. To remove non-miRNA types of non-coding RNAs (e.g., rRNA, tRNA, snRNA, snoRNA), the clean reads were aligned to the Rfam database (v14.9) using Bowtie (v1.3.1). The remaining reads were then mapped to the miRBase v22 database using miRDeep2 (v2.0.1.3) for identification and quantification of known miRNAs.

### 2.6 Bioinformatics analysis

Differential expression analysis was performed using the DESeq2 package (v1.38.3). miRNAs with an absolute log_2_ fold change (FC) ≥1 and an adjusted *p*-value (padj) <0.05 were considered statistically significantly differentially expressed. To identify the downstream regulatory targets of these differentially expressed miRNAs, both predicted and experimentally validated target gene datasets were included in the analysis. Predicted target genes were compiled from multiple established databases and algorithms, including DIANA-microT, ElMMo, MicroCosm, miRanda, miRDB, PicTar, PITA, and TargetScan. To improve prediction accuracy, only target genes concurrently identified by both TargetScan (v7.0) and miRanda (v3.3a) were retained for subsequent analysis. Meanwhile, mature differentially expressed miRNAs (DE miRNAs) were queried in several public databases containing experimentally validated miRNA–target interactions, including miRTarBase (v9.0), miRecords (Release 4), and TarBase (v8.0). These databases curate interactions supported by experimental methods such as qPCR, Western blot, luciferase reporter assays, Northern blot, and high-throughput sequencing.

Subsequently, functional enrichment analysis was performed on the integrated target gene set using the clusterProfiler package (v4.6.2) in R. Enrichment analyses included gene ontology (GO), kyoto encyclopedia of genes and genomes (KEGG), disease ontology (DO), and Reactome pathways, to explore the potential biological functions and disease associations of miRNA-regulated targets.

### 2.7 Quantitative real-time PCR

To further validate the reliability of the high-throughput sequencing results, the top five differentially expressed miRNAs with the highest log_2_ FC values were selected for qRT-PCR (hsa-miR-423-5p, hsa-miR-4689, hsa-miR-2110, hsa-miR-642a-3p, hsa-miR-484). Primers were designed based on cDNA sequences (Table 1). Reverse transcription was performed using the ReverTra Ace qPCR RT Kit (TOYOBO Life Science, China), and qRT-PCR was carried out on a Bio-Rad S1000 system using Bestar SYBR Green Master Mix (TOYOBO). Cycling conditions were as follows: 95°C for 1 min, then 40 cycles of 95°C for 15 s and 60°C for 30 s. Each sample was tested in triplicate. Relative miRNA expression levels were calculated using the 2^−ΔΔCt^ method [22] and normalized to U6 as an internal control.

**Table 1:** Primers used for qRT-PCR

Primer	Sequence (5′−3′)
PG1-24060013-hsa-miR-423-5p (RT)	CTCAACTGGTGTCGTGGAGTCGGCAATTCAGTTGAGAAAGT
PG1-24060013-hsa-miR-423-5p (F)	ACACTCCAGCTGGGGTGAGGGGCAAGAAGCGA
PG1-24060013-hsa-miR-4689 (RT)	CTCAACTGGTGTCGTGGAGTCGGCAATTCAGTTGAGGGCCCCCA
PG1-24060013-hsa-miR-4689 (F)	ACACTCCAGCTGGGTTGAGGACGATGGTG
PG1-24060013-hsa-miR-2110 (RT)	CTCAACTGGTGTCGTGGAGTCGGCAATTCAGTTGAGCACTCAGC
PG1-24060013-hsa-miR-2110 (F)	ACACTCCAGCTGGGTTGGGAAAGCGCC
PG1-24060013-hsa-miR-642a-3p (RT)	CTCAACTGGTGTCGTGGAGTCGGCAATTCAGTTGAGGGTC
PG1-24060013-hsa-miR-642a-3p (F)	ACACTCCAGCTGGGAACATTTGGAGAG
PG1-24060013-hsa-miR-484 (RT)	CTCAACTGGTGTCGTGGAGTCGGCAATTCAGTTGAGATCGGGAG
PG1-24060013-hsa-miR-484 (F)	ACACTCCAGCTGGGTCAGGCTCAGTCCCT

RT: reverse transcription, F: forward.

### 2.8 Statistical analysis

All experiments were independently repeated at least three times, and results are presented as mean value ± standard deviation (SD). Statistical analyses were performed using GraphPad Prism 9 (GraphPad Software, USA) and R software (v4.2.2). Differences between two groups were evaluated using unpaired two-tailed Student’s t-test. A *p*-value <0.05 was considered statistically significant. For miRNA sequencing, differential expression was assessed using the DESeq2 package (v1.38.3) in R, with thresholds of |log_2_ FC| ≥ 1 and padj < 0.05. Functional enrichment analyses (GO, KEGG, DO, Reactome) were conducted using the clusterProfiler package (v4.6.2) in R, with *p*-value adjustment performed using the Benjamini–Hochberg method to control the false discovery rate.

## 3 Results

### 3.1 Determination of the non-cytotoxic concentration of BV

**Figure 1:**
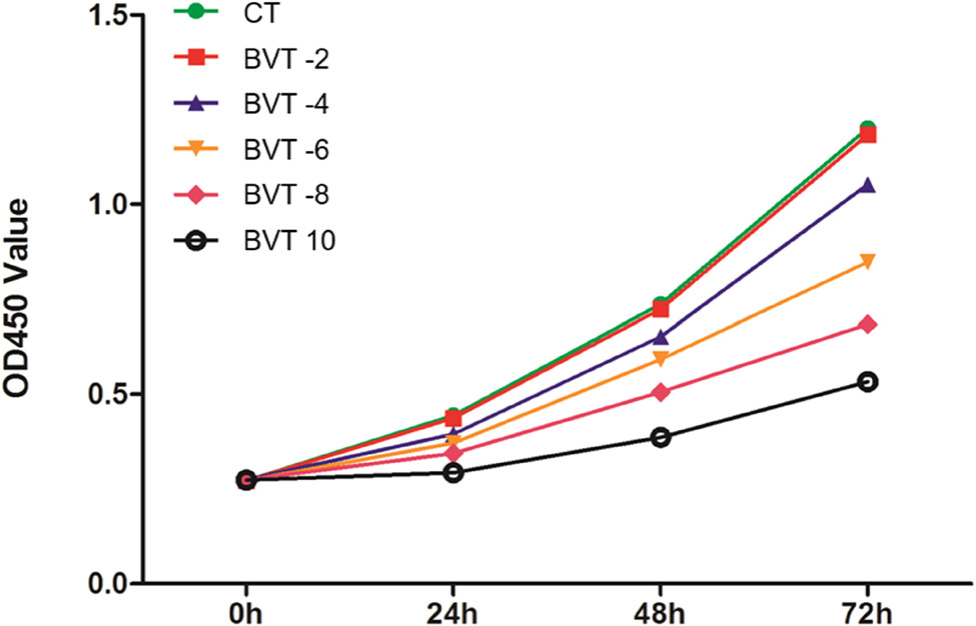
Proliferation capacity of T cells in the CT and BVT groups measured by CCK-8 assay. CT represents T cells, BVT represents BV-treated T cells, and the numbers indicate the corresponding concentrations of BV.

The non-cytotoxic concentration of BV for T cells was determined using the CCK-8 assay (Figure 1). T cells were treated with increasing concentrations of BV (2, 4, 6, 8, and 10 μg/mL). At 2 μg/mL, cell proliferation showed no statistically significant difference compared to the control group. However, concentrations of 4 μg/mL and above led to a marked decrease in proliferation, with 10 μg/mL causing the most substantial inhibition. Based on these results, 2 μg/mL was identified as the non-cytotoxic concentration and was selected for use in subsequent experiments. To allow sufficient accumulation of exosomes for analysis, a 48 h incubation period was chosen for further experiments.

### 3.2 BV enhances exosome secretion in T cells

**Figure 2:**
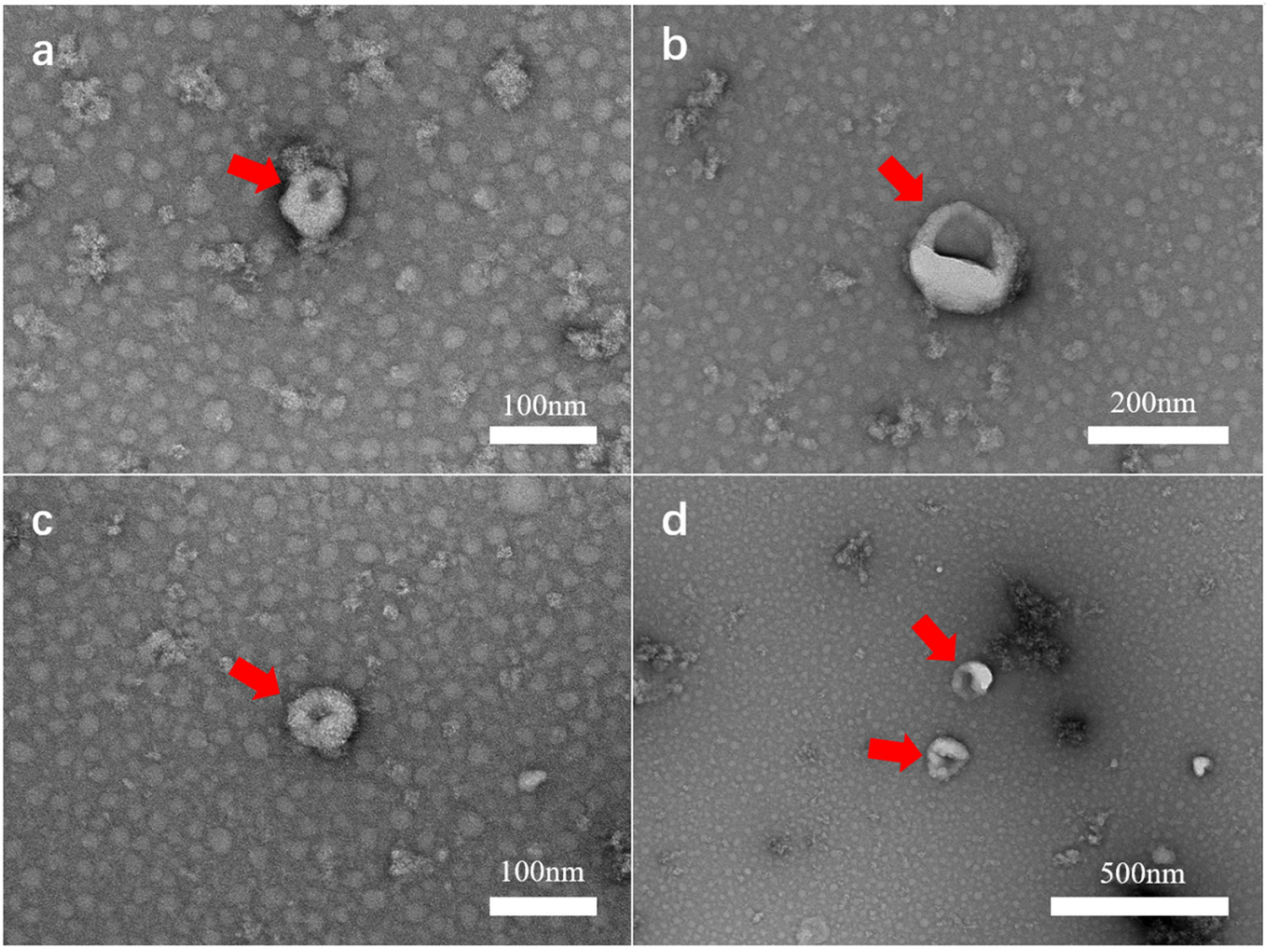
Exosomes under TEM. (a) and (b) Exosomes from BV-treated T cells (BVT-exo). (c) and (d) Exosomes from control T cells (CT-exo). Exosomes are highlighted with red arrows, and the scale bar is marked in the lower right corner of each panel.

**Figure 3:**
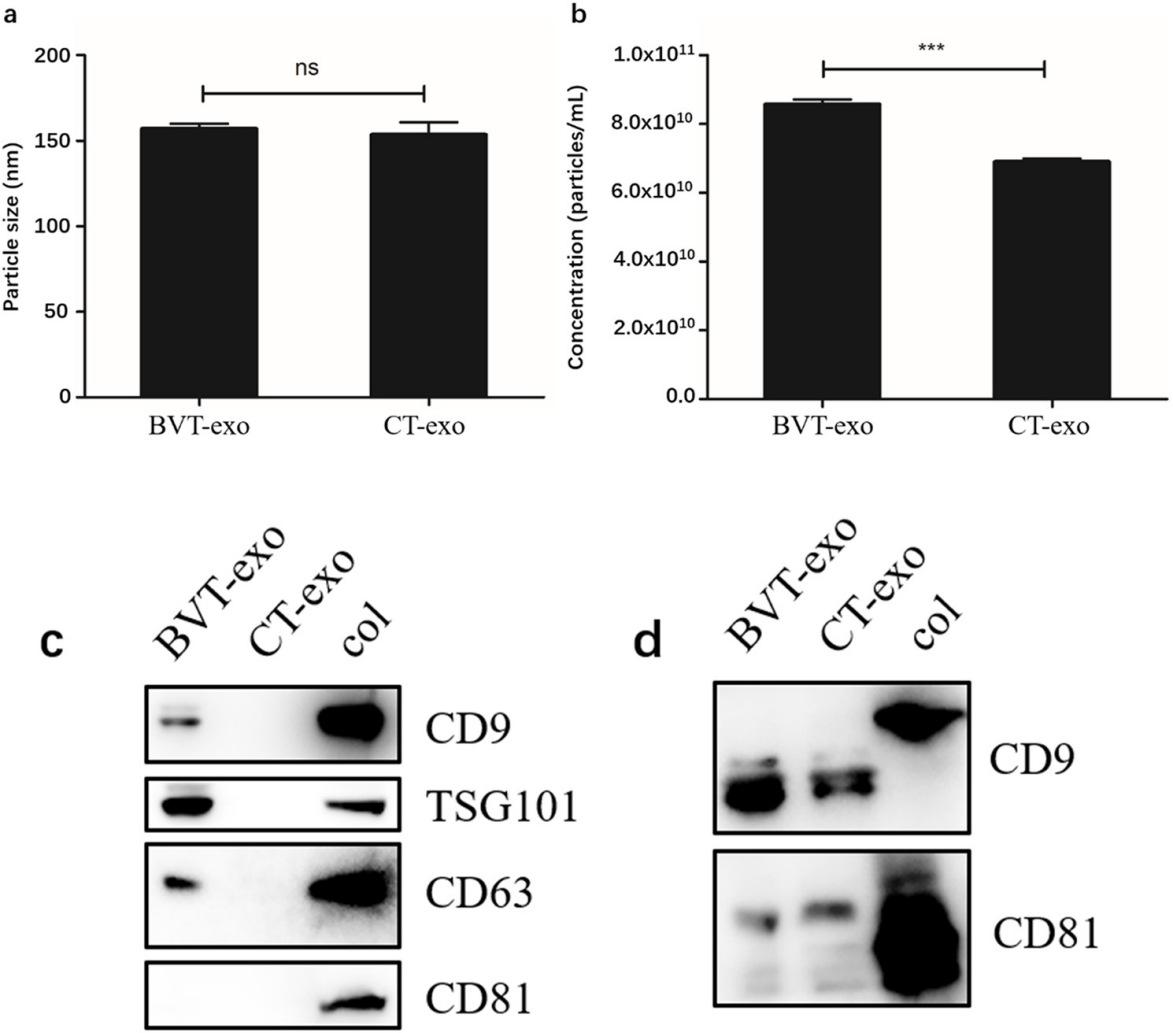
Particle size, concentration, and WB analysis of exosomes. (a) No statistically significant difference in particle size between BVT-exo and CT-exo. (b) BVT-exo concentration is significantly higher than CT-exo, ***P < 0.001, ns, no significant. (c) and (d) Two WB analyses detect characteristic exosomal proteins in both BVT-exo and CT-exo. (c) 20 μg/lane; (d) 40 μg/lane; col: control.

To verify the successful isolation of exosomes, we performed characterization using three complementary techniques. First, TEM revealed the typical cup-shaped morphology of exosomes from both BVT and CT groups, with diameters ranging from 100 to 500 nm (Figure 2). Second, NTA showed average particle sizes of 154.3 nm in the BVT-exo group and 145.2 nm in the CT-exo group, with no statistically significant difference (*P* > 0.05) (Figure 3a), confirming that both fall within the expected size range of exosomes. Third, Western blot analysis detected four canonical exosomal markers – CD9, CD63, CD81, and TSG101 – in both groups (Figure 3c and d), consistent with previously reported findings [23,24].

**Figure 4:**
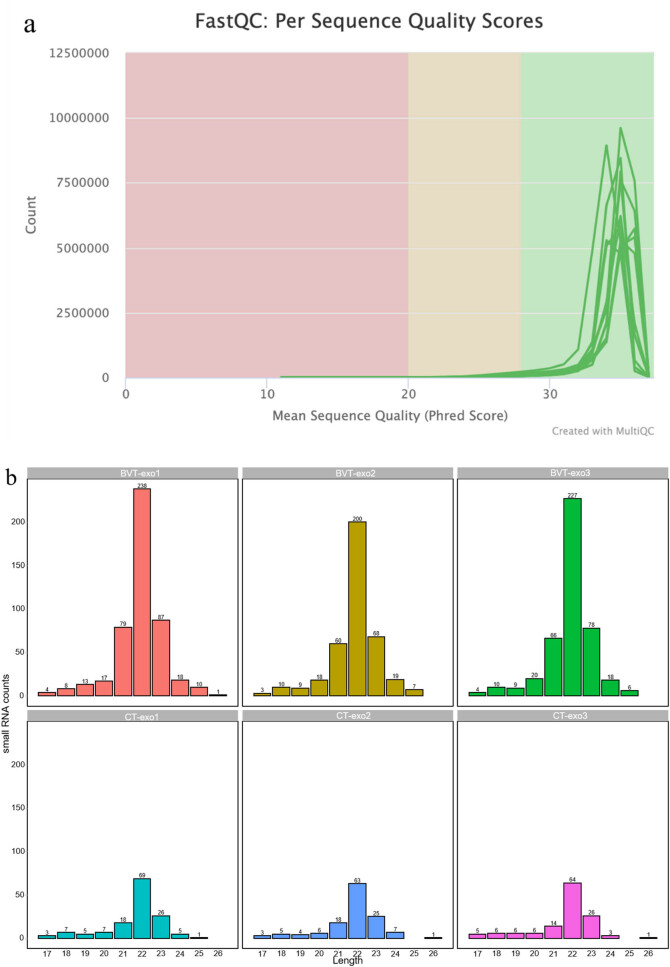

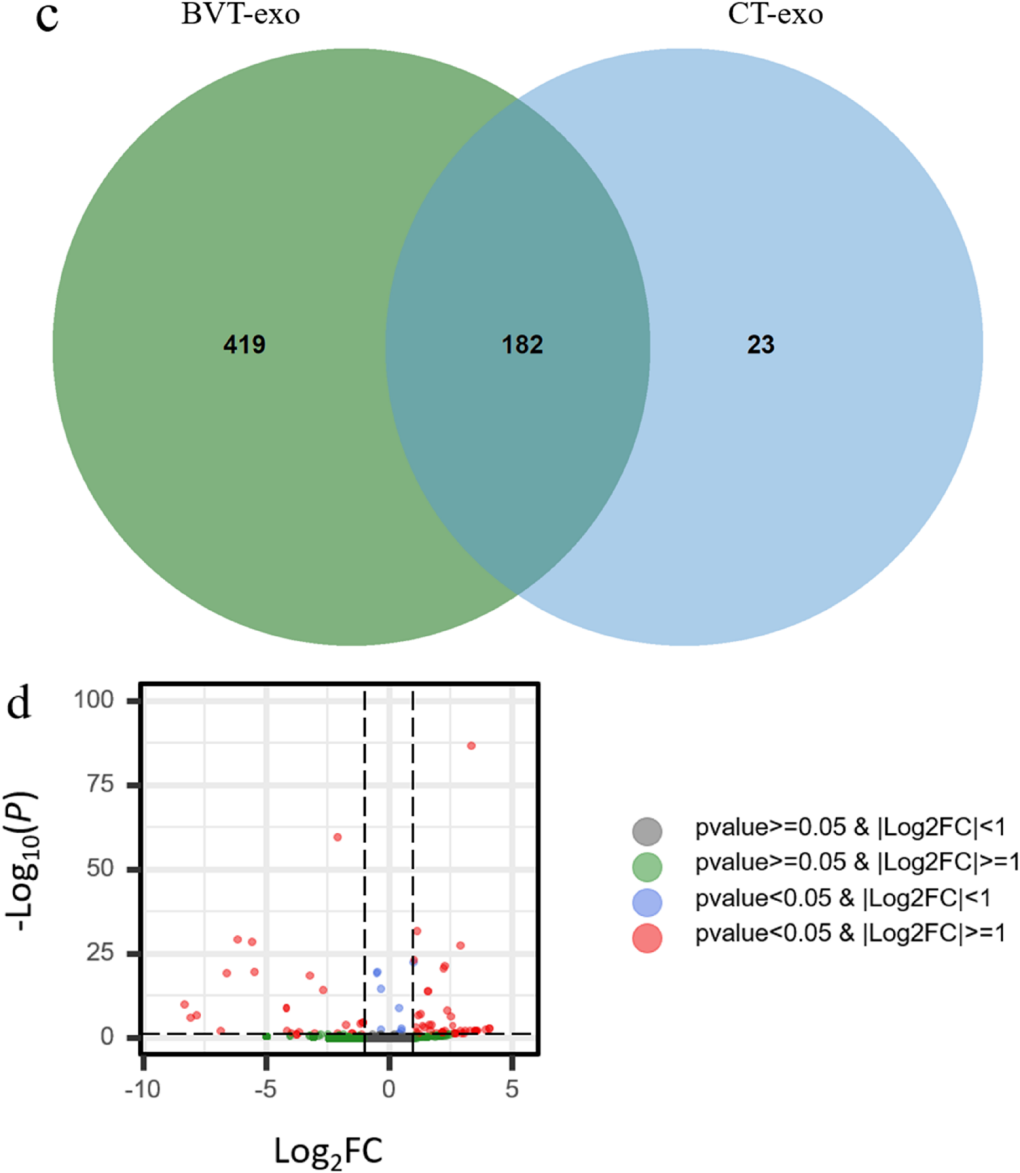
Quality assessment and differential expression analysis of miRNA sequencing data. (a) Distribution of mean sequence quality scores (Phred scores) per read from FastQC. Most reads have high quality scores (>30), indicating good overall sequencing quality. (b) Length distribution of small RNAs in each sample. Small RNAs in the BVT-exo samples are predominantly 22 nucleotides in length, while CT-exo samples show lower overall counts. (c) Venn diagram showing the overlap of identified miRNAs between BVT-exo and CT-exo samples. A total of 182 miRNAs are shared, with 419 unique to BVT-exo and 23 unique to CT-exo. (d) Volcano plot of differentially expressed miRNAs. Color coding is defined in the legend. Threshold: |log_2_ FC| ≥ 1, padj < 0.05.

Importantly, NTA revealed a significantly higher concentration of exosomes in the BVT group (8.4 × 10^10^ particles/mL) compared to the CT group (6.9 × 10^10^ particles/mL), with a highly significant difference (*P* < 0.001) (Figures 3b and 4). These results suggest that BV treatment enhances exosome secretion by T cells. Given this observation, we next examined the miRNA cargo of exosomes.

### 3.3 Upregulated miRNA expression in exosomes from BV-treated T cells

To ensure reliable downstream miRNA analysis, we first assessed the quality of sequencing data. Each sample yielded 12.7–22 million raw reads, of which 6.14–13.94 million passed quality filtering. All samples exhibited Q30 scores above 96% and GC content between 49 and 50%, consistent with typical small RNA libraries (Figure 4a). After removing non-miRNA reads (e.g., rRNA, tRNA, snRNA) via Rfam-based annotation, the remaining reads were mapped to the reference genome, achieving mapping rates of 24–29% and resulting in 1.5–3.79 million mappable reads per sample, which were used for miRNA identification and expression profiling.

The miRNA length distribution was similar between the two groups, with most miRNAs ranging from 17 to 26 nucleotides and peaking at 22 nt. BVT-exo samples contained a higher number of total miRNAs (238, 200, 227) than CT-exo samples (69, 63, 64), suggesting enhanced miRNA loading following BV treatment (Figure 4b). Venn analysis identified 419 miRNAs unique to BVT-exo, 23 unique to CT-exo, and 182 shared between both groups, indicating that BV treatment markedly alters the exosomal miRNA composition (Figure 4c).

Differential expression analysis of exosomal miRNAs between BVT-exo and CT-exo groups identified 749 unique miRNAs. Among them, 74 miRNAs were differentially expressed, including 44 upregulated and 30 downregulated miRNAs (Table 2). The volcano plot revealed a predominance of significantly upregulated miRNAs in BVT-exo, with most displaying high FCs and statistical significance (Figure 4d).

**Table 2:** Differential miRNAs between BVT-exo and CT-exo (ranked by log_2_ FC in descending order)

Number	miRNA	Precursor	Alias	log_2_ FC	padj	Change
1	hsa-miR-423-5p	hsa-mir-423	MIMAT0004748	4.355	<0.001	Up
2	hsa-miR-4689	hsa-mir-4689	MIMAT0019778	4.054	0.001	Up
3	hsa-miR-2110	hsa-mir-2110	MIMAT0010133	4.042	0.001	Up
4	hsa-miR-642a-3p	hsa-mir-642a	MIMAT0020924	3.894	0.003	Up
5	hsa-miR-484	hsa-mir-484	MIMAT0002174	3.552	0.004	Up
6	hsa-miR-193b-5p	hsa-mir-193b	MIMAT0004767	3.522	0.004	Up
7	hsa-miR-3679-5p	hsa-mir-3679	MIMAT0018104	3.505	0.005	Up
8	hsa-miR-361-5p	hsa-mir-361	MIMAT0000703	3.487	0.004	Up
9	hsa-miR-766-5p	hsa-mir-766	MIMAT0022714	3.484	0.005	Up
10	hsa-miR-320a-3p	hsa-mir-320a	MIMAT0000510	3.308	<0.001	Up
11	hsa-miR-296-3p	hsa-mir-296	MIMAT0004679	3.286	0.008	Up
12	hsa-miR-760	hsa-mir-760	MIMAT0004957	3.103	0.004	Up
13	hsa-miR-339-3p	hsa-mir-339	MIMAT0004702	3.029	0.031	Up
14	hsa-miR-6515-5p	hsa-mir-6515	MIMAT0025486	2.952	0.037	Up
15	hsa-miR-877-5p	hsa-mir-877	MIMAT0004949	2.901	<0.001	Up
16	hsa-miR-671-5p	hsa-mir-671	MIMAT0003880	2.865	0.006	Up
17	hsa-miR-92b-3p	hsa-mir-92b	MIMAT0003218	2.692	0.032	Up
18	hsa-miR-505-5p	hsa-mir-505	MIMAT0004776	2.682	0.039	Up
19	hsa-miR-3138	hsa-mir-3138	MIMAT0015006	2.641	0.029	Up
20	hsa-miR-210-3p	hsa-mir-210	MIMAT0000267	2.616	0.034	Up
21	hsa-miR-186-5p	hsa-mir-186	MIMAT0000456	2.571	0.000	Up
22	hsa-miR-629-5p	hsa-mir-629	MIMAT0004810	2.503	<0.001	Up
23	hsa-miR-1307-3p	hsa-mir-1307	MIMAT0005951	2.342	<0.001	Up
24	hsa-miR-185-5p	hsa-mir-185	MIMAT0000455	2.272	<0.001	Up
25	hsa-miR-378a-3p	hsa-mir-378a	MIMAT0000732	2.245	0.004	Up
26	hsa-miR-130b-3p	hsa-mir-130b	MIMAT0000691	2.183	<0.001	Up
27	hsa-miR-9-3p	hsa-mir-9-1	MIMAT0000442	2.133	0.014	Up
28	hsa-miR-9-3p	hsa-mir-9-2	MIMAT0000442	2.133	0.014	Up
29	hsa-miR-9-3p	hsa-mir-9-3	MIMAT0000442	2.133	0.014	Up
30	hsa-miR-18a-5p	hsa-mir-18a	MIMAT0000072	1.906	0.032	Up
31	hsa-miR-95-3p	hsa-mir-95	MIMAT0000094	1.704	0.000	Up
32	hsa-miR-625-5p	hsa-mir-625	MIMAT0003294	1.685	0.006	Up
33	hsa-miR-17-5p	hsa-mir-17	MIMAT0000070	1.595	<0.001	Up
34	hsa-miR-92a-3p	hsa-mir-92a-2	MIMAT0000092	1.571	<0.001	Up
35	hsa-miR-92a-3p	hsa-mir-92a-1	MIMAT0000092	1.554	<0.001	Up
36	hsa-miR-421	hsa-mir-421	MIMAT0003339	1.433	0.000	Up
37	hsa-miR-342-3p	hsa-mir-342	MIMAT0000753	1.345	0.000	Up
38	hsa-miR-196a-5p	hsa-mir-196a-1	MIMAT0000226	1.284	<0.001	Up
39	hsa-miR-744-5p	hsa-mir-744	MIMAT0004945	1.173	0.022	Up
40	hsa-miR-196a-5p	hsa-mir-196a-2	MIMAT0000226	1.154	<0.001	Up
41	hsa-let-7b-5p	hsa-let-7b	MIMAT0000063	1.106	<0.001	Up
42	hsa-miR-30d-5p	hsa-mir-30d	MIMAT0000245	1.100	0.011	Up
43	hsa-miR-532-5p	hsa-mir-532	MIMAT0002888	1.065	0.000	Up
44	hsa-miR-16-5p	hsa-mir-16-2	MIMAT0000069	1.002	<0.001	Up
45	hsa-miR-181a-5p	hsa-mir-181a-1	MIMAT0000256	−1.059	<0.001	Down
46	hsa-miR-181a-5p	hsa-mir-181a-2	MIMAT0000256	−1.059	<0.001	Down
47	hsa-miR-122-5p	hsa-mir-122	MIMAT0000421	−1.140	0.045	Down
48	hsa-miR-15b-5p	hsa-mir-15b	MIMAT0000417	−1.154	<0.001	Down
49	hsa-miR-29b-3p	hsa-mir-29b-1	MIMAT0000100	−1.528	0.021	Down
50	hsa-miR-29b-3p	hsa-mir-29b-2	MIMAT0000100	−1.528	0.021	Down
51	hsa-miR-12136	hsa-mir-12136	MIMAT0049032	−1.754	<0.001	Down
52	hsa-miR-155-5p	hsa-mir-155	MIMAT0000646	−2.090	0.029	Down
53	hsa-miR-4516	hsa-mir-4516	MIMAT0019053	−2.107	<0.001	Down
54	hsa-miR-4488	hsa-mir-4488	MIMAT0019022	−2.697	<0.001	Down
55	hsa-miR-1290	hsa-mir-1290	MIMAT0005880	−2.927	0	Down
56	hsa-miR-328-3p	hsa-mir-328	MIMAT0000752	−3.012	0.027	Down
57	hsa-miR-619-5p	hsa-mir-619	MIMAT0026622	−3.234	<0.001	Down
58	hsa-miR-10401-5p	hsa-mir-10401	MIMAT0041633	−3.671	0.017	Down
59	hsa-miR-1-3p	hsa-mir-1-1	MIMAT0000416	−3.784	0.048	Down
60	hsa-miR-1-3p	hsa-mir-1-2	MIMAT0000416	−3.784	0.048	Down
61	hsa-miR-5585-3p	hsa-mir-5585	MIMAT0022286	−3.989	0.019	Down
62	hsa-miR-222-5p	hsa-mir-222	MIMAT0004569	−4.144	<0.005	Down
63	hsa-miR-3648	hsa-mir-3648-1	MIMAT0018068	−4.181	<0.001	Down
64	hsa-miR-3648	hsa-mir-3648-2	MIMAT0018068	−4.181	<0.001	Down
65	hsa-miR-1246	hsa-mir-1246	MIMAT0005898	−5.386	0	Down
66	hsa-miR-10400-5p	hsa-mir-10400	MIMAT0041631	−5.484	<0.001	Down
67	hsa-miR-3960	hsa-mir-3960	MIMAT0019337	−5.573	<0.001	Down
68	hsa-miR-4508	hsa-mir-4508	MIMAT0019045	−6.179	<0.001	Down
69	hsa-miR-4492	hsa-mir-4492	MIMAT0019027	−6.607	<0.001	Down
70	hsa-miR-1471	hsa-mir-1471	MIMAT0007349	−6.870	0.004	Down
71	hsa-miR-7704	hsa-mir-7704	MIMAT0030019	−6.916	<0.001	Down
72	hsa-miR-3196	hsa-mir-3196	MIMAT0015080	−7.830	<0.001	Down
73	hsa-miR-4484	hsa-mir-4484	MIMAT0019018	−8.089	<0.001	Down
74	hsa-miR-4787-5p	hsa-mir-4787	MIMAT0019956	−8.322	<0.001	Down

Note: Log fold change = log_2_ FC; threshold: |log_2_ FC| ≥ 1, padj < 0.05.

### 3.4 qRT-PCR validation of sequencing

**Figure 5:**
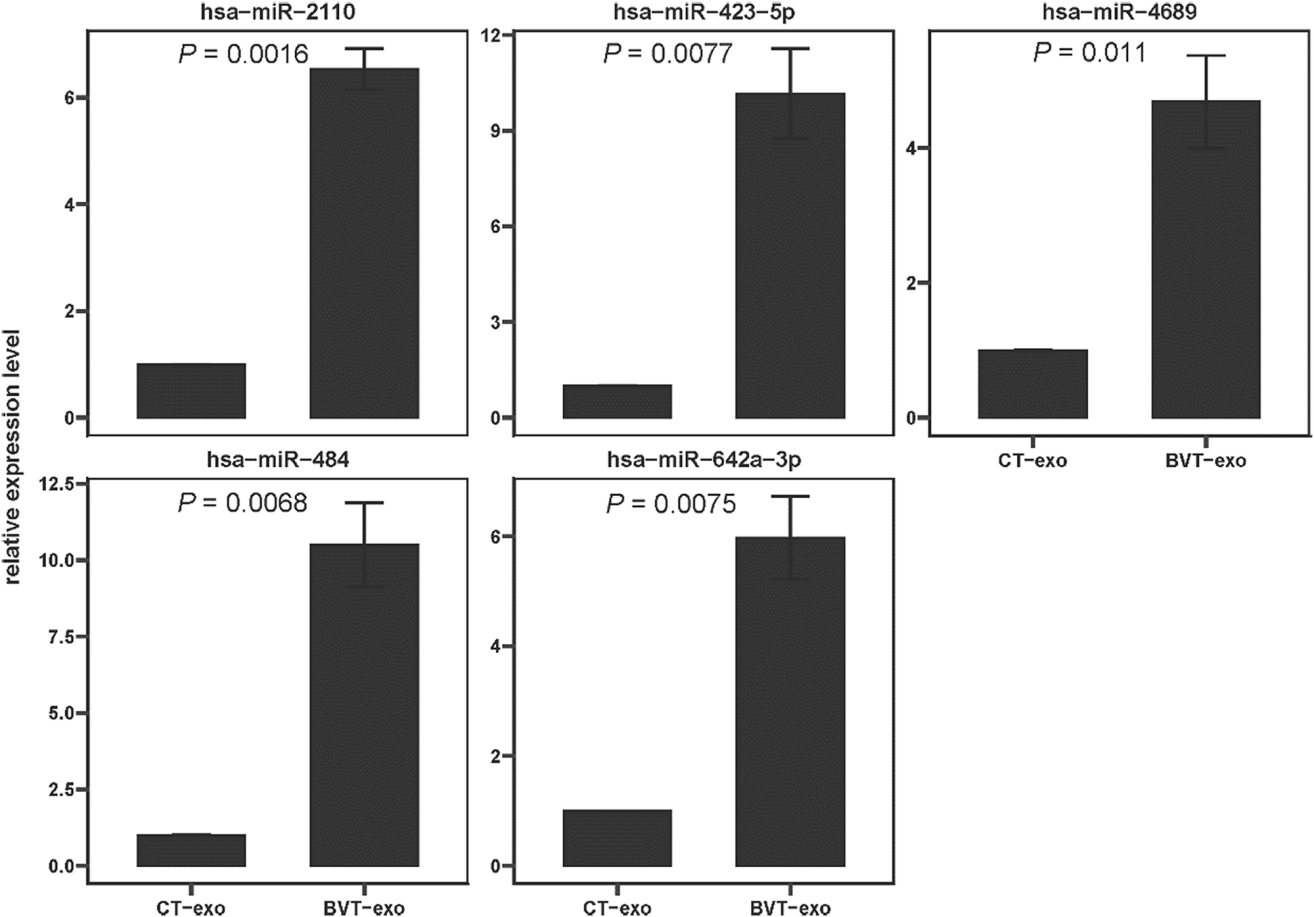
qRT-PCR validation of differentially expressed miRNAs identified by high-throughput sequencing. Data are presented as mean value ± standard deviation (*n* = 3). *P* < 0.05 was considered statistically significant.

To validate the high-throughput sequencing results, qRT-PCR was performed to assess the expression of five representative upregulated miRNAs. As shown in Figure 5, the expression levels of hsa-miR-2110, hsa-miR-423-5p, hsa-miR-4689, hsa-miR-484, and hsa-miR-642a-3p were significantly higher in exosomes derived from BVT-exo compared to CT-exo, with *P* values of 0.0016, 0.0077, 0.011, 0.0068, and 0.0075, respectively. These qRT-PCR results were consistent with the sequencing data, confirming that BV treatment leads to the selective upregulation of specific miRNAs in T cell–derived exosomes.

### 3.5 Functional enrichment analysis of target genes of DE miRNAs

**Figure 6:**
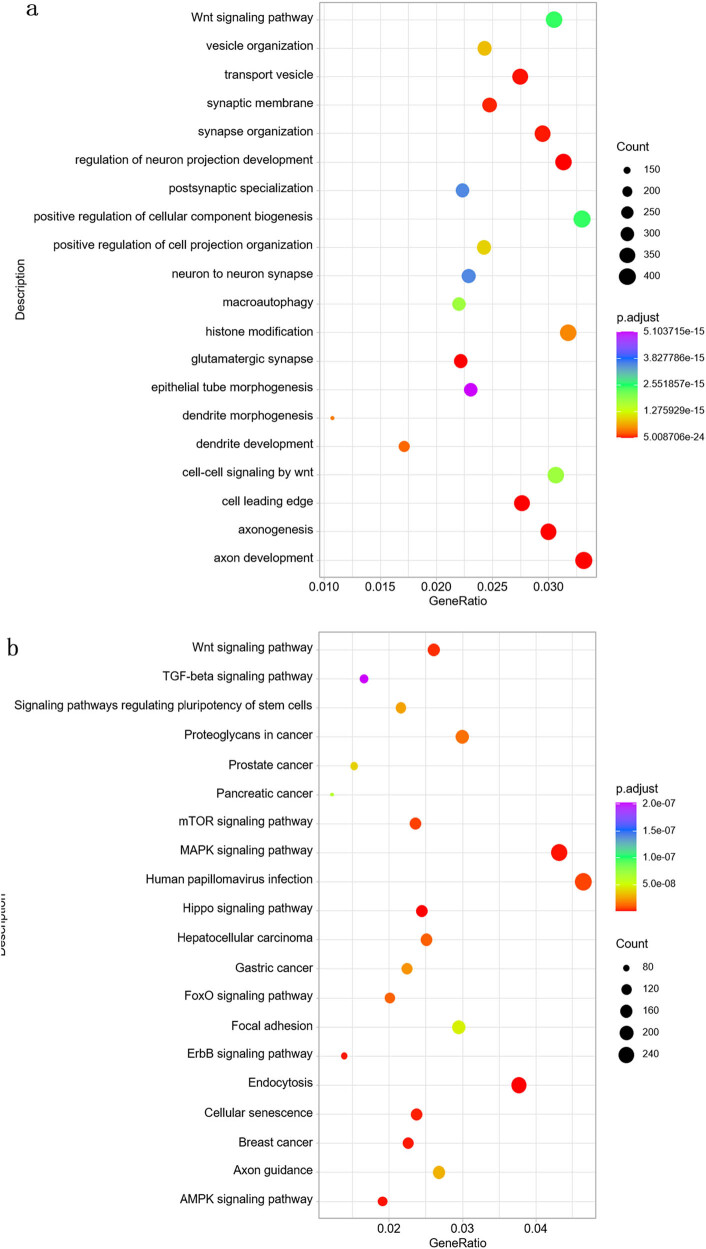

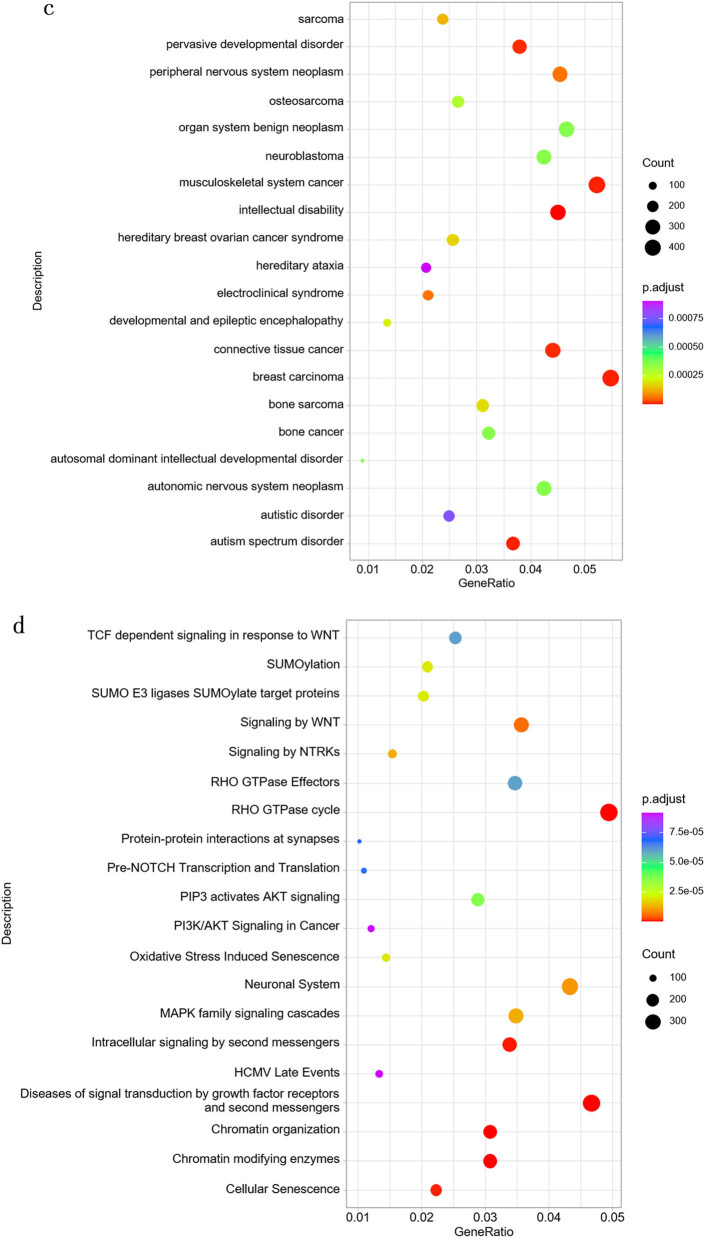
Functional enrichment of target genes predicted from differentially expressed miRNAs. (a) GO: Primarily involves neuron-related biological processes. (b) KEGG: Mainly includes classic signaling pathways such as Wnt, TGF-beta, and MAPK, with particular relevance to pancreatic, prostate, and gastric cancers. (c) DO: Differential genes are closely associated with neurological developmental disorders and cancer-related diseases. (d) Reactome: Involves various cellular signaling pathways and processes. Threshold: |log_2_ FC| ≥ 1, padj < 0.05.

To explore the biological roles of DE miRNAs, we performed target gene prediction followed by pathway enrichment analysis. GO analysis showed enrichment in neurodevelopment-related processes, including the Wnt signaling pathway, synaptic membrane structure, synapse organization, neuron projection development, and postsynaptic specialization (Figure 6a). KEGG pathway analysis indicated significant enrichment in signaling pathways such as Wnt, TGF-β, MAPK, and mTOR, as well as in several cancer-associated pathways (Figure 6b). DO analysis linked these target genes to a wide range of neurological and oncological conditions, including autism spectrum disorder, intellectual disability, neuroblastoma, osteosarcoma, and breast carcinoma (Figure 6c). Reactome pathway analysis further highlighted roles in Wnt and PI3K/AKT signaling, chromatin remodeling, SUMOylation, and related pathways (Figure 6d).

**Figure 7:**
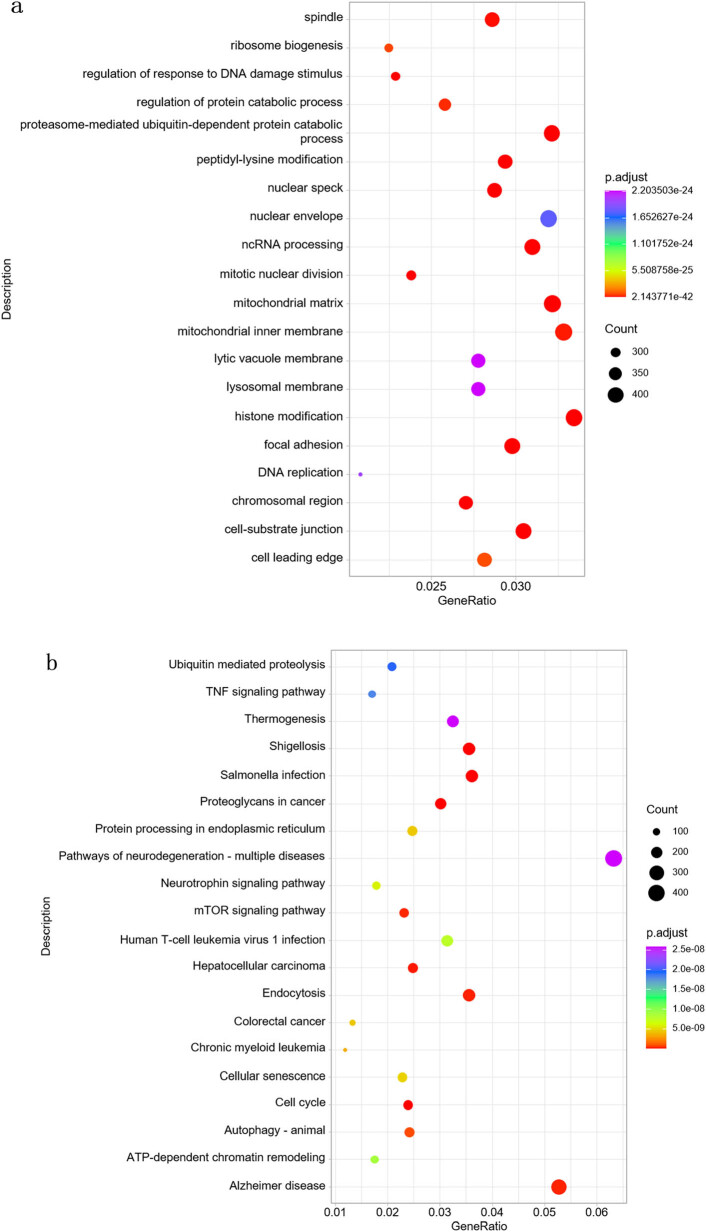

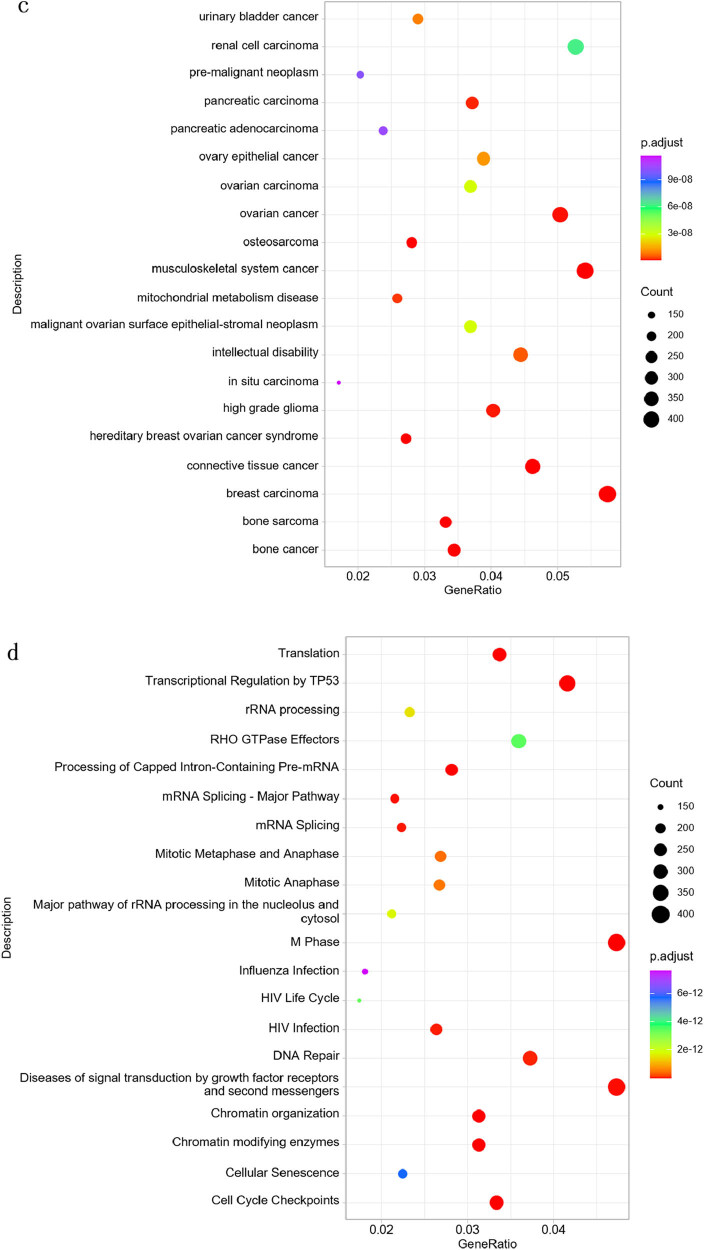
Functional enrichment of validated target genes of differentially expressed miRNAs. (a) GO: Significant processes include spindle formation, ribosome biogenesis, DNA damage response regulation, protein degradation, and mitochondrial-related processes. (b) KEGG: Involves ubiquitin-mediated protein degradation, TNF signaling pathway, mTOR signaling pathway, and cancer-related pathways such as hepatocellular carcinoma and colorectal cancer. (c) DO: Includes disease types such as PC, ovarian cancer, osteosarcoma, urothelial cancer, and mitochondrial metabolic disorders. (d) Reactome: Mainly enriched in transcription, TP53-regulated transcription, RHO GTPase cycles, and related pathways. Threshold: |log_2_ FC| ≥ 1, padj < 0.05.

To enhance the reliability of functional interpretation, we also analyzed only experimentally validated target genes. GO enrichment revealed involvement in cell cycle regulation, chromatin modification, protein catabolism, and DNA damage response mechanisms (e.g., spindle assembly, ribosome biogenesis, and ubiquitin-proteasome pathways) (Figure 7a). KEGG analysis confirmed enrichment in ubiquitin-mediated proteolysis, TNF/mTOR signaling, neurotrophin pathways, and disease pathways such as Alzheimer’s disease, colorectal cancer, and chronic myeloid leukemia (Figure 7b). DO analysis identified associations with bladder, renal, pancreatic, and ovarian cancers, as well as mitochondrial metabolic disorders and intellectual disability (Figure 7c). Reactome analysis emphasized roles in transcriptional regulation (e.g., TP53 signaling), mRNA processing, chromatin remodeling, and DNA repair (Figure 7d).

Together, these analyses suggest that DE miRNAs in BVT-derived exosomes may participate in critical biological processes, including cell proliferation, genetic stability, neural development, and oncogenesis. The high degree of consistency between predicted and validated target analyses supports the hypothesis that these miRNAs may serve as key regulators and potential biomarkers in both neurological and cancer-related diseases.

## 4 Discussion

In this study, we explored the effects of BV on exosome secretion and miRNA cargo in human T cells. Our results show that BV, at a non-cytotoxic concentration of 2 µg/mL, significantly promotes exosome release and alters the miRNA composition of exosomes derived from Jurkat T cells. Notably, the BV-induced exosomal miRNAs were enriched in pathways related to cell proliferation, neurodevelopment, and cancer, indicating a potential role for BV in modulating intercellular communication via exosomal signaling.

A key observation of this study is that BV treatment significantly enhances exosome secretion released by T cells, as confirmed by NTA, without affecting their size distribution or morphology. The presence of upregulated exosomal markers in both control and BV-treated groups supports the successful isolation and characterization of these vesicles. This finding aligns with previous reports demonstrating that external stimuli – such as oxidative stress, inflammatory signals, and certain drugs – can modulate exosome biogenesis [25,26,27]. Our results suggest that components of BV may act as biological stimuli that activate intracellular pathways involved in exosome production and release. A second major finding is that BV treatment led to marked alterations in the miRNA profiles of exosomes. A total of 74 miRNAs were differentially expressed between the BV-treated and control groups, with the majority showing upregulation. This shift suggests that BV may regulate the selective sorting of miRNAs during exosome formation. Although the underlying mechanisms remain to be fully elucidated, previous studies have proposed that cellular stress or immune activation can promote the selective packaging of specific miRNAs into exosomes via pathways involving the ESCRT complex or RNA-binding proteins such as hnRNPA2B1 and YBX1 [28,29]. It is plausible that BV induces similar cellular responses, thereby facilitating the enrichment of functionally relevant miRNAs within exosomes.

Functional enrichment analyses of the predicted and validated target genes of differentially expressed miRNAs revealed biological processes and signaling pathways closely associated with T cell function and disease (Figures 6 and 7). GO and KEGG analyses identified significant enrichment in pathways related to cell cycle regulation, synapse organization, and Wnt, MAPK, and TGF-β signaling – processes essential for immune regulation, neuronal development, and oncogenesis. Notably, the BV-induced miRNAs were also enriched in pathways implicated in neurological disorders and various cancers, including neuroblastoma, colorectal cancer, and breast carcinoma. These findings suggest a dual role for BV-induced exosomal miRNAs in both immune modulation and disease progression. Among the most upregulated miRNAs, hsa-miR-423-5p, hsa-miR-484, and hsa-miR-2110 have been previously linked to tumorigenic processes. For instance, hsa-miR-423-5p has been shown to promote cell proliferation in breast and gastric cancers [30,31], while hsa-miR-484 is associated with angiogenesis and mitochondrial function [32,33]. These miRNAs may serve as potential biomarkers of BV exposure or as therapeutic targets in BV-based interventions. However, whether these miRNAs exert similar functions within the context of exosome-mediated intercellular communication remains to be clarified and warrants further investigation.

Several limitations should be acknowledged in this study. First, while we focused on the changes in exosomal miRNAs following BV treatment in T cells, other exosomal components such as proteins and long non-coding RNAs were not investigated, and their potential functions remain unclear. Second, BV consists of multiple bioactive components, each of which may regulate distinct biological processes. Future studies using individual components such as melittin or PLA2 are needed to dissect their specific mechanisms. Third, this study used the Jurkat T cell line, which has limited physiological relevance; validation in primary T cells is necessary to assess the generalizability of our findings. Fourth, although we predicted the potential targets and functions of miRNAs through bioinformatic analyses, functional validation is lacking. Future work should evaluate the biological effects of BV-induced exosomes using assays for cytokine production, cell proliferation, and migration.

In conclusion, our study found that BV enhances exosome secretion in T cells and significantly alters their miRNA composition. These changes may influence pathways related to immune signaling, neurodevelopment, and cellular regulation. This work offers preliminary clues for further investigation into how BV regulates exosome function; however, the specific mechanisms and biological significance remain to be clarified.
